# Palestine-Israel War Coping Strategies of Tunisian People

**DOI:** 10.1192/j.eurpsy.2024.565

**Published:** 2024-08-27

**Authors:** N. Messedi, F. Guermazi, A. Samet, I. Chaari, M. Sehli, F. Charfeddine, L. Aribi, J. Aloulou

**Affiliations:** ^1^Psychiatry B, Hedi Cheker University Hospital, Sfax, Tunisia

## Abstract

**Introduction:**

The war in Gaza is a stressful life event. Due to its significant human and financial losses, it affected the mental health of people around the world including the middle east citizens.

**Objectives:**

To study the coping strategies of Tunisian people toward Palestine-Israel war in its first month and the factors associated with them.

**Methods:**

It was a cross-sectional, descriptive and analytical study, conducted among Tunisians. Data were collected during October and November 2023, through an anonymous online questionnaire, spread throughout social media (Facebook/Instagram), using the Google Forms® platform. We used a socio-demographic and clinical data sheet and the “Brief-COPE” to assess coping strategies.

**Results:**

A total of 1091 participants completed the questionnaire. Their mean age was 32,7± 9.8 years, with a sex-ratio (F/M) of 3.5. Among participants, 46,1% are married, 42,5% have children and 19,5% have a psychiatric follow history. Sport’s practitioners represent 23,3% of the participants and 10,6% increased their use of sports after the war news.

In terms of coping strategies: problem focused coping was the most used strategy (mean= 2,02) followed by emotional focused coping (mean= 1,98) and avoidant coping (mean= 1,63). Tunisians rely the most on religion, accepting reality and planning as coping mechanisms (score= 2,85; 2,4 and 2,23 respectively). Substance use was the last resort option (score= 1,11).

Our survey revealed significant associations between coping mechanisms and several factors: Venting, humor and behavioral disengagement were significantly correlated with sex gender (p=0,000 ; 0,000 ; 0,000 respectively); Substance use coping mechanism was significantly correlated with participants having a psychiatric follow history (p=0,001); Avoidant coping subscale was significantly correlated with having children (p=0,000); Self distraction was significantly correlated with the increase use of sport among Tunisians (p=0,000).

**Conclusions:**

These findings underscore the need for healthcare and productive coping strategies for Tunisians and middle east people during the Palestine-Israel war.

**Disclosure of Interest:**

None Declared

